# Ultrafast Electronic Coupling Estimators: Neural Networks versus Physics-Based Approaches

**DOI:** 10.1021/acs.jctc.3c00184

**Published:** 2023-06-22

**Authors:** Roohollah Hafizi, Jan Elsner, Jochen Blumberger

**Affiliations:** Department of Physics and Astronomy and Thomas Young Centre, University College London, Gower Street, London WC1E 6BT, United Kingdom

## Abstract

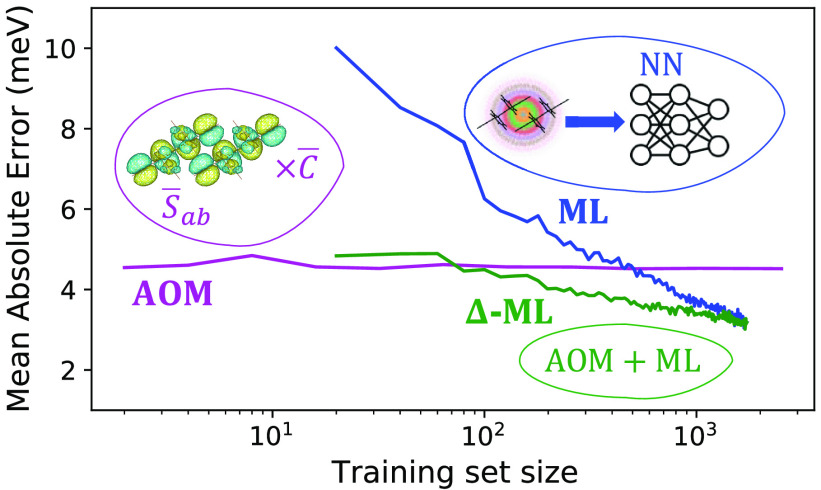

Fast and accurate estimation of electronic coupling matrix elements between molecules is essential for the simulation of charge transfer phenomena in chemistry, materials science, and biology. Here we investigate neural-network-based coupling estimators combined with different protocols for sampling reference data (random, farthest point, and query by committee) and compare their performance to the physics-based analytic overlap method (AOM), introduced previously. We find that neural network approaches can give smaller errors than AOM, in particular smaller maximum errors, while they require an order of magnitude more reference data than AOM, typically one hundred to several hundred training points, down from several thousand required in previous ML works. A Δ-ML approach taking AOM as a baseline is found to give the best overall performance at a relatively small computational overhead of about a factor of 2. Highly flexible π-conjugated organic molecules like non-fullerene acceptors are found to be a particularly challenging case for ML because of the varying (de)localization of the frontier orbitals for different intramolecular geometries sampled along molecular dynamics trajectories. Here the local symmetry functions used in ML are insufficient, and long-range descriptors are expected to give improved performance.

## Introduction

Charge transport simulations in biology and materials science typically begin with the calculation of electronic coupling matrix elements, or transfer integrals.^1−5^ There have been significant advances in computing electronic couplings in the last 20 years. Depending on the requirements of the problem at hand, a large number of techniques are now available. The choice of method is dictated by various factors, most importantly by the right balance between accuracy and the associated computational cost. A number of approaches can be employed to accomplish this task: from high accuracy yet expensive ab initio calculations^6−8^ to density functional theory (DFT) calculations (e.g., time-dependent DFT,^9^ constrained DFT,^10−15^ projector operator-based diabatization,^16−18^ fragment-orbital DFT,^19,20^ and frozen density embedding),^21,22^ to fast semiempirical density functional tight binding (DFTB),^7,8,23^ to the analytic overlap method (AOM).^24,25^

For a typical simulation of charge carrier transport in soft condensed media (e.g., organic and biological semiconductors) using, e.g., Kinetic Monte Carlo (KMC),^26−29^ transient localization theory,^30,31^ or non-adiabatic molecular dynamics (NAMD) simulations,^32−35^ a very large number of transfer integrals must be evaluated before the simulation of charge mobility is converged. Some time ago, our group introduced the analytic overlap method (AOM), an ultrafast approach for the calculation of electronic coupling matrix elements for electron transfer between π-conjugated molecules. AOM allows one to estimate couplings to a useful degree of accuracy and about 10^5^ times faster than with DFT calculations.^24^ This method proposes to substitute the computationally expensive calculation of charge transfer integrals by an efficient calculation of the frontier molecular orbital (FMO) overlap integrals, multiplied by a suitable linear scaling coefficient. The FMOs are constructed using an optimized Slater-type orbital (STO) basis set, allowing ultrafast analytical calculations of FMO overlap integrals and electronic couplings for a variety of dimers.^24,36^

While AOM predicts electronic couplings to a useful degree of accuracy for applications in, e.g., KMC or NAMD^32^ simulations, they are associated with an error because the relation between overlap and coupling is not strictly linear and the data exhibit a fair amount of scatter (see, e.g., Figure 2). Apart from this, challenging cases for AOM are flexible molecules that may adopt configurations that lead to significant changes in the localization/delocalization of the FMO. In this case the expansion coefficients of the FMOs have to be reoptimized using expensive DFT calculations, which is not desirable. Besides, there may be distinct dimers in a unit cell with significantly different chemical interactions, necessitating multiple linear scaling constants. One such case, discussed below, is O-IDTBR, a molecule that belongs to the class of non-fullerene acceptors, a promising molecule for the organic photovoltaics industry. The purpose of this study is to explore the potential of atomic neural networks for machine learning of electronic couplings and for error estimation of the physics-based AOM (Δ-ML).

Various machine learning methods have been used to model the electronic coupling between molecular pairs. Musil et al.^37^ used the Gaussian process (GP) to predict electronic couplings between rigid molecules based on their relative positions and orientations. A similar approach was taken by Lederer et al.,^38^ who employed kernel ridge regression (KRR) to target rigid molecules. In a study by Bag et al.,^39^ feature vectors were extracted from DNA via a coarse-grained model, and a neural network was trained to evaluate electronic couplings. Wang et al.^40^ and Caylak et al.^41^ used Coulomb matrices (CMs) as the molecular descriptor for training GP and deep neural networks, respectively. Also, Miller et al.^42^ tried many ML methods, compared their performances, and suggested random forests as the most effective method. There are a number of shortcomings in previous ML models, including either a lack of accuracy in predictions, the necessity to freeze some degrees of freedom of the system, or the requirement for a large number of training (reference) data. We aim in this paper to address all of these issues by “semi-physical” modeling of electronic couplings and to compare our model’s performance to that of previous models. The term “semi-physical” refers to the fact that we approximate electronic couplings as the sum of atomic contributions which are modeled by neural networks. At the current stage, ML models predict couplings only between chemically identical molecules, but in arbitrary atomic configurations. In other words, the ML model is not intended to make predictions for molecules other than those for which it has been trained but has the potential to be generalized.

**Figure 1:**
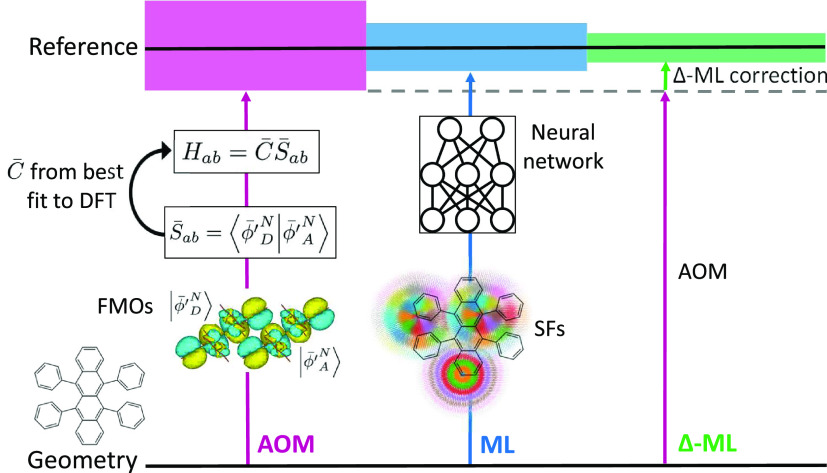
Three models are used in this work to calculate electronic coupling values between dimer molecules: (AOM, purple) The electronic overlap between approximated frontier molecular orbitals is determined, and utilized to estimate electronic couplings between dimer molecules. (ML, blue) The coordinates of dimer atoms are used to calculate the symmetry function and estimate the reference coupling value. (Δ-ML, green) A neural network is employed to correct the AOM model estimations to the reference values. To optimize the number of training points for each model, a few data sampling methods are studied.

In the following, after a short introduction to the methods, a protocol is developed for sampling reference data points required for the ML model that ensures completeness of sampling with the least number of data points. Figure 1 shows a schematic of the methods used in this work to predict electronic couplings. Two neural network models of electronic couplings of dimers are then trained on (1) DFT reference data points (Figure 1, blue) and (2) the difference between DFT reference data points and AOM (Figure 1, green) in a process called Δ-ML. Results are compared to those from the AOM model (Figure 1, purple). Using a rubrene dimer data set, these models are compared in terms of their performance. We then use the best model to study a challenging molecule for electronic coupling estimation, O-IDTBR. As a point of clarification, throughout the text, the terms “electronic coupling” and “transfer integral” are synonymous.

## Methods

The AOM method assumes a linear relationship between electronic coupling *H*_*ab*_ of two diabatic wavefunctions, ψ_*a*_ and ψ_*b*_,
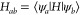
1where *H* is the electronic Hamiltonian, and the corresponding wavefunction overlap,
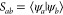
2such that
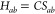
3Full calculation of eq 2 is computationally expensive, since it requires explicit diabatic wave functions ψ_*a*_ and ψ_*b*_, for example, approximated by Kohn–Sham determinants obtained from constrained density functional theory.^15^ Instead, the assumption is made that charge transport is mediated solely by the frontier molecular orbitals (FMOs) of the isolated molecules, *ϕ′*_*D*_^*N*^ and *ϕ′*_*A*_^*N*^ (notation as in refs (24) and (25)). In the case of hole (electron) transport, these will correspond to the HOMO (LUMO) orbitals of the molecules. To further increase the efficiency of calculations, the FMOs are expressed in a minimum Slater-type orbital (STO) basis. The FMO of a single representative molecule, *ϕ′*_*l*_^*N*^ (*l* = *D* or *A*), is calculated from an explicit DFT calculation once and is projected onto a minimal STO basis to yield ϕ̅′_*l*_^*N*^:
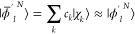
4where |χ_*k*_⟩ is the *k*th STO orbital and *c*_*k*_ is the corresponding expansion coefficient. This allows for an ultrafast analytic calculation of the orbital overlap *S̅*_*ab*_ = ⟨ϕ̅′_*D*_^*N*^|ϕ̅′_*A*_^*N*^⟩ , since the overlap of STO basis functions is known analytically. Further details concerning the DFT calculation and projection to the STO basis can be found in refs (24) and (25). Importantly, orbital expansion coefficients are kept constant for different dimer geometries, while the direction of STO orbitals is updated according to the geometry of the molecule. The validity of this final approximation depends on the extent to which the localization/delocalization of the FMO is preserved for different molecular configurations and will be discussed further below. To account for our representation of the FMO in the STO basis, eq 3 is rewritten as
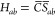
5where *C̅* is obtained from a best fit of *S̅*_*ab*_ to *H*_*ab*_ reference data computed at the explicit electronic structure level, typically DFT.

Unlike previous attempts at ML of electronic couplings, which map dimer descriptors in the input layer to a single value in the output, our approach approximates the total electronic coupling *J*_*ij*_ as the sum of contributions of atoms in monomer *i* and monomer *j*:
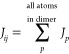
6This is in analogy to the AOM, where orbital overlap is calculated as a sum of overlap contributions from all atom pairs. Second-generation neural network potentials^43^ use a similar approach to approximate energy. This method is included in the open-source code n2p2^44^ and is used to train our neural network models. Each element (H, C, etc.) has a network, and atoms of the same element have the same weights and biases. The neural network of each element consists of two hidden layers, each with 20 nodes. Following the notation in ref (45), the neural network architecture is *N*–20–20–1, where *N* is the length of the atomic local-environment descriptor. The functional form of a neural network of an element is given by eq 7, and atoms in the same atom types share the same weights and biases. Each element (H, C, etc.) has a separate neural network with the following functional form:
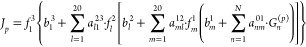
7in which *f*’s are activation functions (we used *f*^1^(*x*) = *f*^2^(*x*) = tanh(*x*) and *f*^3^(*x*) = *x*), *a*_*kl*_^*ij*^ is the weight connecting node *k* in layer *i* to node *l* in layer *j*, and *b*_*j*_^*i*^ is the bias attached to node *j* in layer *i*. The values of the *a*_*kl*_^*ij*^ and *b*_*j*_^*i*^ are fitted during the training process. After training the network, the weights and biases remain fixed for all predictions.

At the heart of the function defined in eq 7 lies the *N*-dimensional structural descriptor vector:
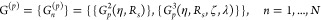
8The structural descriptors convert each dimer’s atomic structure into a rotation-, translation-, and permutation-invariant input for the neural network. Within a cutoff radius of 8 Å, each atom’s local environment is described by atom-centered symmetry functions (SFs). The radial environment of each atom is captured using radial symmetry functions:^44,46^
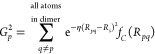
9where atom *p* is the central atom for which the symmetry function is calculated, *R*_*s*_ is the shift in the center of the Gaussian peak, η is the width of Gaussians, and *f*_*C*_(*R*_*pq*_) is the cutoff function:
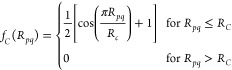
10In order to obtain a better description of the atomic environment, radial symmetry functions are calculated for all element doublets (CC, CH, HC, HH, etc.) We use eight radial symmetry functions for each pair of elements, whose η and *R*_*s*_ parameters are determined using the method introduced by Imbalzano et al.^47^ Thus, there are 8 × *N*_*E*_ radial symmetry functions for each atom in a system with *N*_*E*_ elements. For each element triplet, angular functions of the following form are generated to describe the angular environment of each atom:^44,46^
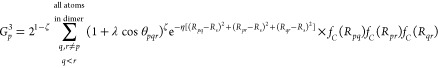
11where atom *p* is the central atom and θ_*pqr*_ is the angle formed by atoms *p*, *q*, and *r*. Like radial symmetry functions, angular symmetry functions are calculated for all element triplets (CCC, CCH, CHH, etc.) to better describe the environment around the atoms. For each element triplet, there are two *R*_*s*_ values, two ζ values, and two λ values, resulting in eight angular symmetry functions that are automatically selected.^47^ As there are *N*_*E*_(*N*_*E*_ + 1)/2 element triplets, each atom will have 8 × *N*_*E*_(*N*_*E*_ + 1)/2 angular descriptors.

A total of 3612 rubrene dimer geometries were taken from an ab initio molecular dynamics trajectory using the optPBE-vdW density functional,^48^ a DZVP basis set,^49^ and GTH pseudopotentials.^50^ Snapshots from four distinct dimer pairs within a supercell of 12 molecules were taken at 50 fs intervals. Further computational details can be found in ref (51). A total of 5770 O-IDTBR dimer pairs were taken from classical molecular dynamics trajectories using a force field parametrized specifically for the family of IDTBR non-fullerene acceptors.^52^ All reference DFT electronic couplings were calculated using the projector-operator-based diabatization (POD) method^17^ in conjunction with the Perdew–Burke–Ernzerhof (PBE) density functional and a uniform scaling constant of 1.325. This is referred to as the sPOD/PBE method. The scaling factor was obtained from the best fit to ab initio reference values for the HAB79 database of organic dimers.^53^

## Results and Discussion

### Optimal Sampling Protocol for AOM Fitting

As a starting point, we present a protocol for fitting the AOM. A representative collection of molecular dimers is sampled from molecular dynamics trajectories to fit the linear scaling relation between AOM overlap and sPOD/PBE electronic coupling (eq 5). Depending on the complexity of the physical system, there may be hundreds to thousands of points sampled.^25,32,51^ For crystalline systems, the sampled dimers are arranged in a few clusters that contain fairly similar dimers. Dimers within a cluster may have a wide range of electronic couplings, despite being visually similar. Consequently, chemical intuition cannot easily determine whether the dimer space is undersampled or oversampled. Undersampling of the data limits the validity of the model, whereas oversampling of the data increases the cost of reference electronic coupling calculations. A further consequence of oversampling data is that it may result in inconsistent AOM scaling constants depending on which part of the dimer space has been oversampled. As a means of addressing these issues, we propose a method of systematic sampling that ensures convergence of the scaling factor with a small number of samples.

**Figure 2:**
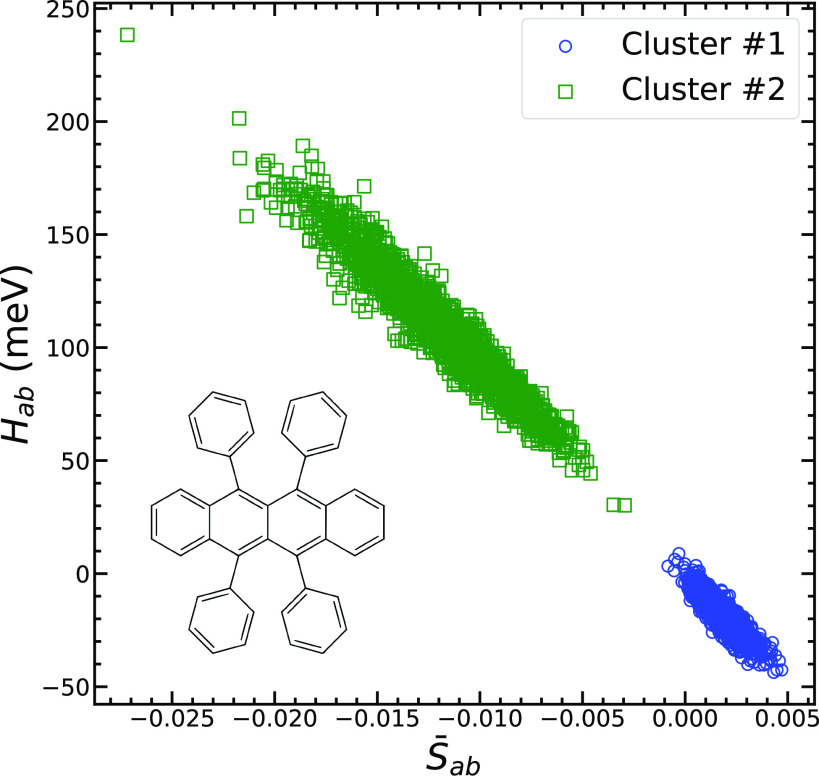
Clustering of the rubrene DFT reference data set using hdbscan and AMD descriptor. Two clusters are detected and shown as green squares and blue circles, corresponding to orbital overlap (*S̅*_*ab*_) and electronic coupling (*H*_*ab*_) in rubrene dimers along the crystallographic directions *a* and *b*, respectively.

From a geometrical perspective, the AOM reference data set should represent all possible dimers. It is therefore necessary to use a geometrical descriptor that is capable of accurately capturing the similarity of the structures in order to select the most different structures. In this study, we utilize the average minimum distance (AMD),^54^ a geometrical descriptor that has recently been proposed. AMD is rotation-, translation-, and permutation-invariant; it is also stable and computationally efficient. As a test of its quality, we clustered rubrene dimers extracted from an ab initio MD simulation trajectory for a rubrene molecular crystal at room temperature.^51^ The result of clustering using the hdbscan clustering algorithm^55^ are shown in Figure 2.

In hdbscan, the distance metric is the Euclidean distance between AMD descriptors. The data set contains 3612 data points arranged in two clusters with size 1806. These two clusters represent the electronic couplings in the *a* and *b* crystallographic directions, respectively; they are shown by circles and squares in Figure 2. Using the AMD descriptor, hdbscan divides the data set into two clusters indicated by the green and blue colors. It is important to note that no data points remain unclustered and that both clusters are detected correctly without any errors, indicating that AMD is a good descriptor to capture the underlying order of the data.

**Figure 3:**
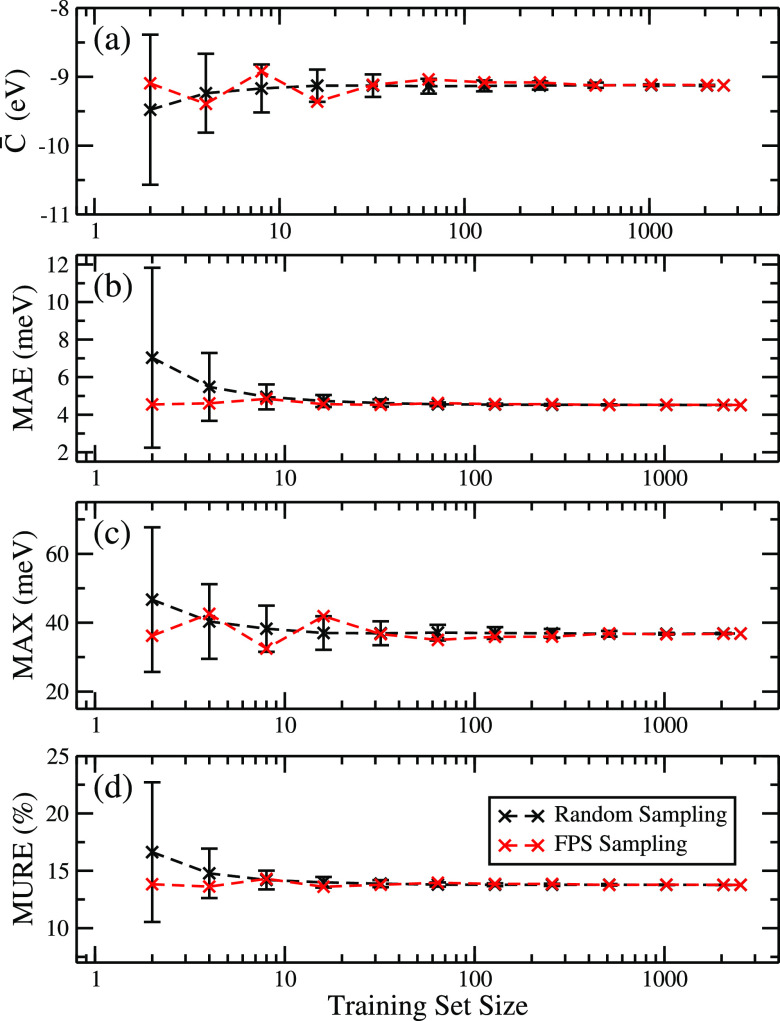
Convergence of AOM scaling constant *C̅* in rubrene and error metrics with training set size: (a) the scaling constant of the AOM model, (b) the mean absolute error of predictions on the test set, (c) the maximum error of predictions, and (d) the mean unsigned relative error of predictions using either random sampling (black) or the FPS algorithm (red). An error bar indicates one standard deviation based on 10 randomly selected training sets of the corresponding size.

To sample the reference data points for AOM fitting, AMD is used as a descriptor, and the farthest point sampling (FPS) algorithm^56,57^ is applied. This is an iterative optimization strategy that selects the most diverse data points from already-selected data. With a good descriptor, such as AMD, the dimer configurational space will be sampled in a uniform manner such that oversampling and undersampling are prevented. This allows us to sample dimers with the greatest geometric diversity with the smallest number of data points. Figure 3a illustrates the convergence of fitting the AOM’s scaling factor, *C̅*, with respect to the size of the training set when data points are sampled randomly (black) or by AMD+FPS (red). As the number of data points sampled by FPS is increased from 32 to 64, the fitted *C̅* value differs by less than 1%, and the value can be considered converged for any practical purposes. The *C̅* value obtained from random sampling depends strongly on the particular sample chosen, especially when training set sizes are small. In Figure 3a we plot the average *C̅* value obtained from 10 random samples for each training set size, and the root-mean-square deviation of the *C̅* value across these 10 samples is indicated by error bars. The data show that random sampling can lead to large errors and cannot guarantee a reliable *C̅* value, while FPS is very robust, even at small sample sizes, and provides an optimal sampling strategy. The completeness of the sampled set is also verified by the convergence of the mean absolute error (MAE), the maximum absolute error (MAX), and the mean unsigned relative error (MURE) in Figure 3b–d. Errors were calculated over 30% of the data that was not used for sampling the training set for fitting *C̅*. The protocol for optimal sampling for AOM is summarized as follows:

1. Run a long-enough MD simulation, on the order of 100 ps to 1 ns.
2. Sample dimers at a frequency which accurately samples the fluctuations of electronic couplings, typically on the order of 100 fs for molecular crystals.
3. Sort the initial data set by FPS using AMD geometrical descriptor.
4. Pick *n* new data points from the sorted list, calculate the reference ab initio electronic couplings, and include them in the AOM reference data.
5. Fit the scaling constant of AOM, *C̅*.
6. Repeat steps 4 and 5 until the change in *C̅* is less than 1%.

Using this method, the smallest set of structures with maximum geometrical diversity is collected for AOM, thereby saving a considerable amount of computational time.

### Machine Learning Models

**Figure 4:**
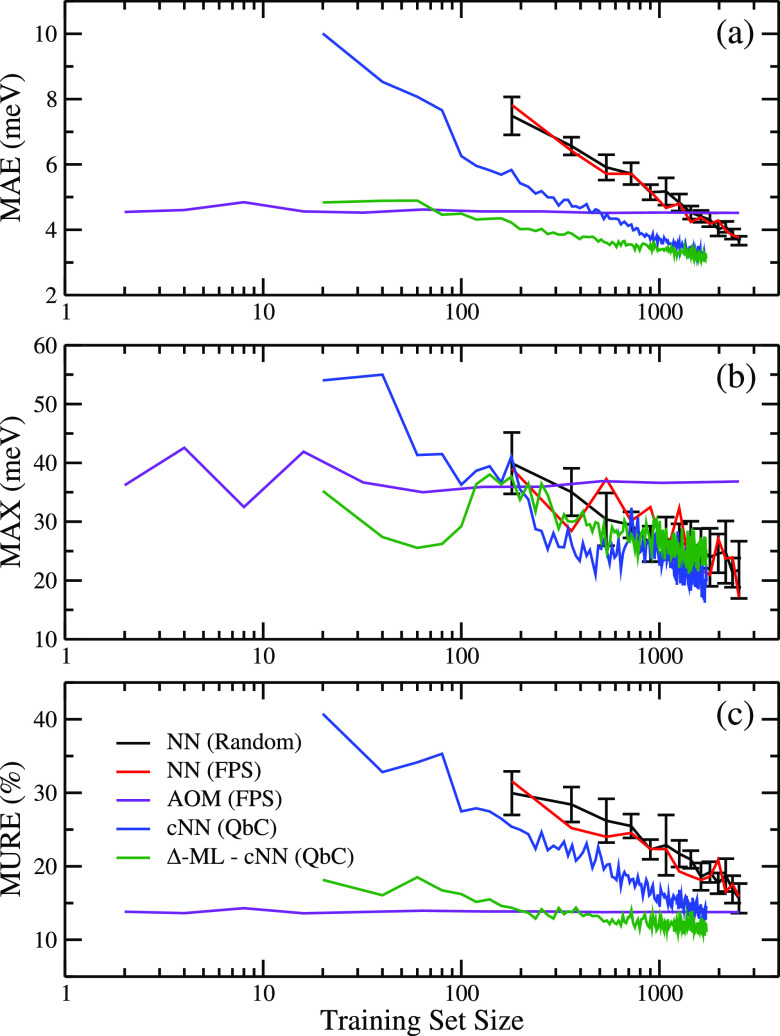
ML of electronic couplings of rubrene dimers. (a) MAE, (b) MAX, and (c) MURE vs training set size when electronic couplings are evaluated by AOM model (purple), a neural network with randomly sampled training set (black), a neural network with FPS-sampled training set (red), a QbC-sampled committee neural network (cNN) (blue), and a QbC-sampled cNN trained on the difference between reference data and AOM values (green).

While FPS is a good sampling method for selecting data to parametrize the AOM, it is not the best algorithm for preparing the training set for neural network (NN) models where larger amounts of data are required compared to the AOM. This was determined by comparing the learning curve of a neural network model for electronic couplings between rubrene dimers when the training set was sampled randomly versus when it was sampled by FPS, as described in the previous section. This comparison is shown in Figure 4 and indicates only a very slight difference between random sampling (black) and FPS sampling (red). FPS sampling was also performed with atomic descriptors based on symmetry functions, and the results were minimally improved compared to random sampling (see Figure S2). In addition to having no advantage over random sampling, FPS sampling also results in larger errors when training sets are small. Consequently, sampling training sets based on geometrical diversity does not necessarily result in improved neural networks. It is evident that both the black and red learning curves show that the NN is capable of achieving a higher level of accuracy than the AOM model (Figure 4, data in purple), in particular with regard to the MAX error, which is reduced by a factor of about 2. However, a rather large reference data set of more than 1000 DFT electronic couplings is needed to outperform AOM. While for rubrene dimers this is computationally manageable, for larger molecules or for systems with a larger number of nearest-neighbor couplings, a more data efficient NN method is desirable.

Active learning methods, in particular committee neural networks (cNN),^58^ can often provide greater data efficiency. For this purpose, a committee of *N* neural networks are trained on slightly differing training sets in order to sample different parts of the hyperdimensional space of neural network weights. The committee is used to make predictions on data that have not been incorporated into the training set. Those data points with the highest level of disagreement, as measured by the standard deviation of the committee’s prediction, are added to the training set. This so-called query by committee (QbC) process is repeated iteratively until the disagreement between committee members on the pool of unseen data converges to that of the training set. Detailed information about the approach can be found in the work of Schran et al.^58^

In this study, we utilize a committee of eight neural networks in order to learn the electronic couplings of rubrene dimers; 70% of the data is used for training, and 30% is used for testing. Initially, 20 dimers are selected randomly from the training pool. Each committee member is trained on 80% of this data (16 data points out of 20), such that each committee member sees a slightly different training set. The committee members are applied to the remainder of the training pool, and the 20 structures with the highest disagreement between committee members are added to the committee training pool. This procedure is repeated iteratively until the disagreement between committee members on the training pool and training set data converges. We differ from the work of Schran et al.^58^ in that our disagreement score is based on the standard deviation of predicted electronic couplings.

Query by committee takes 2–3 times less data to beat AOM in terms of MAE than random sampling (Figure 4, blue lines). In addition, the cNN method allows us to achieve lower MAE than random sampling: ∼3.2 meV with around 1600 data points selected by the cNN versus ∼3.8 meV when all the training data are included. This indicates that there is hidden redundancy in the data set that biases the model to specific dimer configurations when all of the data are used. The MAX and MURE also decrease faster when QbC is used.

The linear relationship between electronic coupling and orbital overlap shown in Figure 2 indicates that the underlying assumptions of the AOM are a good approximation for this system. However, there is still some scatter. In a Δ-ML approach, we use AOM as a baseline and attempt to learn the difference between DFT electronic coupling and AOM values (scatter or deviation from linear relation) using neural networks. This approach has proven very successful when there is a high correlation between a computationally inexpensive baseline and the target accuracy.^59^ AOM provides an excellent baseline for this purpose due to its high correlation with the reference data, *R*^2^ = 0.992, as shown in Figure 2.

A cNN model is trained on the difference between DFT electronic couplings and the AOM value (Δ-ML) using the iterative QbC sampling approach described above. The learning curves are shown in Figure 4 (green lines); about an order of magnitude less data than using standard cNN is required to outperform AOM, a significant improvement. Furthermore, with only 500 dimers used for training, this is the only model that achieves a better MURE (∼11%) than AOM (∼14%). It is worth noting that if the test set includes many reference values close to zero, the MURE will be very large, since small absolute error translates into a large relative error. This is less of a problem for AOM, since the assumed AOM relation eq 5 passes through the origin. We therefore benefit from using AOM as a baseline in order to improve the MURE of predictions.

In the current work, the electronic coupling has been approximated as a sum of atomic contributions, which is the most straightforward approximation if one writes the density in terms of a sum of atomic densities (with any partitioning algorithm). The works of Wang et al.^40^ and Caylak et al.^41^ are similar in this regard, as both use Coulomb matrices^60^ (CMs) as descriptors. CMs describe a system via inverse distances between atoms but do not include higher-order terms. This method is fast to compute and easy to implement, and it allows the reconstruction of an atomistic system using a least-squares approach.^60^ Although it uses atomic numbers directly to encode elements, it suffers from discontinuities in the sorted version or from information loss in the diagonalized version since its eigenspectrum is not unique. We instead assign different symmetry functions to different element pairs and triplets depending on their type, both for pairwise radial (*G*^2^) and triplewise angular (*G*^3^) symmetry functions. The use of such descriptors allows for a more accurate description of the chemical environment and thus for a faster and better training of the corresponding model. As opposed to the findings of Musil et al.,^37^ we demonstrated that active learning by QbC is a more successful sampling strategy than FPS, at least when neural networks are the ML method of choice. As shown in Figures 4 and S2, the use of FPS did not provide any significant improvement over random sampling when geometrical descriptors, such as AMD and SFs, were used as the input.

### A Challenging Case: O-IDTBR

A case study is presented to illustrate the use of the presented machine learning approach to estimate electronic couplings in O-IDTBR. This material belongs to the class of non-fullerene acceptors (NFAs), which have recently attracted significant interest in organic photovoltaics (OPVs). Their contribution to record OPV efficiencies (currently 19%) results from a number of inherent, desirable NFA characteristics, such as synthetically tunable optical properties, improved long-term morphological stability, and high charge carrier mobility (μ).^61,62^ In comparison with other NFAs, O-IDTBR exhibits a superior structure/packing motif, resulting in a relatively high electron mobility for this class of materials.^63^

**Figure 5:**
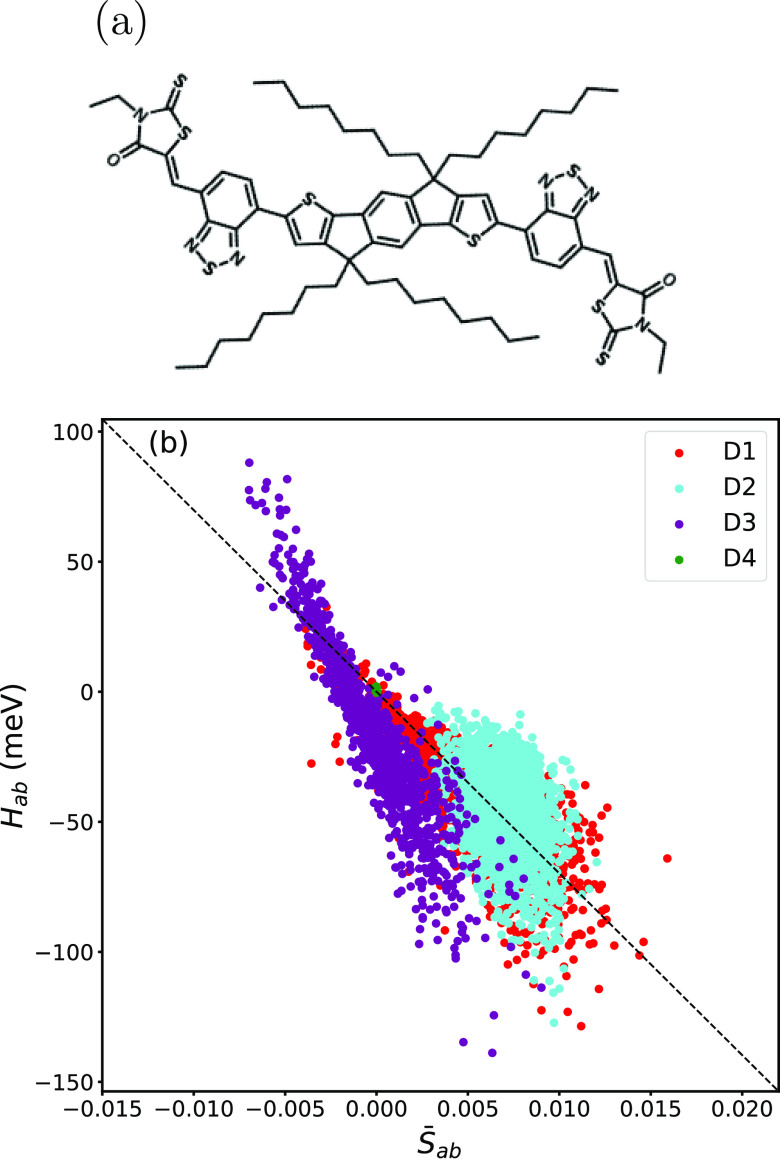
(a) Chemical structure of O-IDTBR. (b) Scatter plot of electronic coupling from DFT calculations (sPOD method) vs orbital overlap from reconstructed FMOs with fixed expansion coefficients. Calculations were carried out for dimers extracted from classical MD simulation of the O-IDTBR crystal. D1 to D4 denote different dimer orientations in the crystal structure.

A total of 5770 dimer configurations were taken from classical molecular dynamics trajectories using a force field specifically parametrized for the family of IDTBR NFAs.^52^ This data set contains four distinct dimer types, the structures of which are shown in Figure S4. Figure 5 shows electronic coupling versus orbital overlap for the data set, where overlap values were calculated using the fixed expansion coefficients of the O-IDTBR molecule at the minimum-energy configuration. Different dimer types are labeled by color. Due to a large center-of-mass distance between monomers, overlaps and electronic couplings for D4 dimers are close to zero. Clearly, the deviation from the linear relationship between overlap and coupling is now very significant, and the scatter is much larger than for rubrene. Moreover, it appears that the data would be better described by two slopes, one for dimer geometries labeled D3 and another for the other dimer types. The physical reason for the large scatter is discussed below. We would expect ML models to give a more significant improvement over AOM than for rubrene.

**Figure 6:**
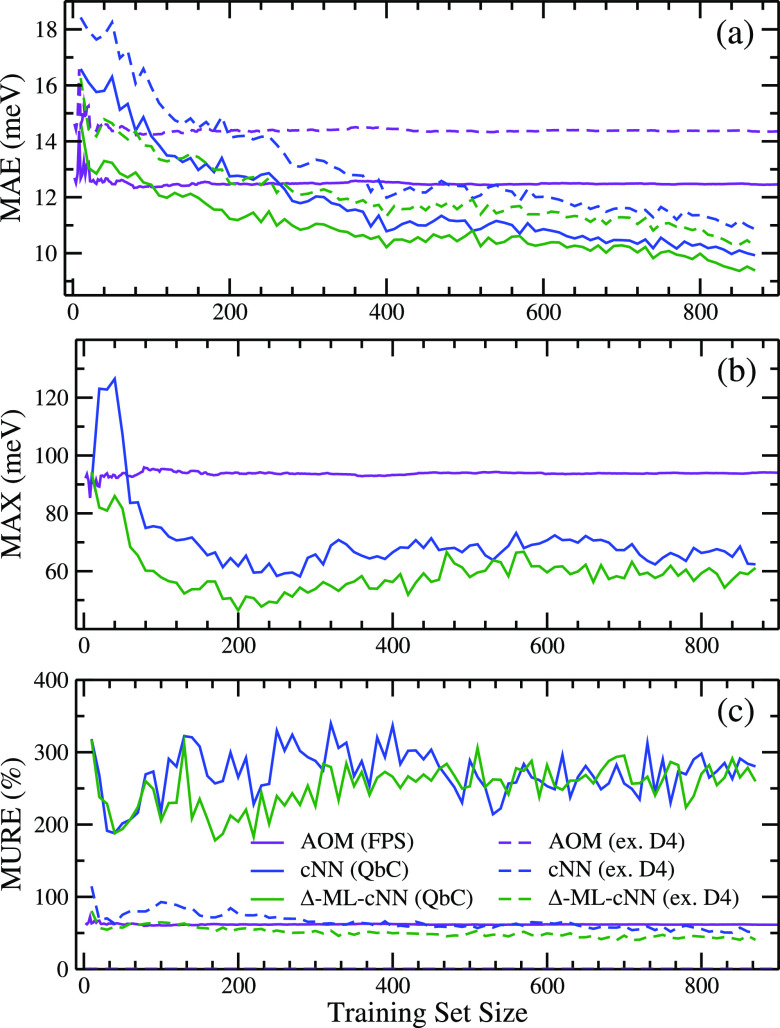
ML of electronic couplings for O-IDTBR. (a) MAE, (b) MAX, and (c) MURE vs training set size when electronic couplings are evaluated by AOM model (purple), a QbC-sampled committee neural network (blue), and a QbC-sampled committee neural network trained on the difference between reference data and AOM values (green). Solid and dashed lines represent test sets that include and exclude D4 dimers.

We started by testing the AMD descriptor’s ability to assign molecular dimers to one of the four dimer types. We used the hdbscan algorithm, AMD descriptors, and Euclidean distances as described previously, results of which are presented in Figure S5. Once again, clustering is successful without any errors or unclustered data points. By randomly assigning 5270 data points to the training set and the remaining 500 data points to the test set, we applied the sampling protocol introduced previously to fit the AOM model. A converged *C̅* value of −7070 meV was obtained after adding 200 dimers to the training set (see Figure S7). This model results in an MAE of 12.4 meV, a MAX of 94 meV, and a MURE of 62% on the test set (see Figure 6, purple lines).

Figure 5 clearly shows that the correlation between the AOM and reference couplings (*R*^2^ = 0.67) is less strong than for rubrene (*R*^2^ = 0.99). We train cNN models using both direct learning and Δ-learning as described previously. The parameters for the atomic environment descriptors, i.e., symmetry functions, are the same as those for rubrene. O-IDTBR consists of five chemical elements, with each element having 40 radial and 120 angular symmetry functions. Therefore, the dimension of the descriptor is 3 times larger than for rubrene. In order to reduce the computational costs, we first pruned all symmetry functions with values below 10^–4^. We then trained a simple model on a small subset of the data set and carried out a sensitivity analysis to remove those symmetry functions for which the output layer of the NN is less than 0.4% sensitive to their gradients. Accordingly, we have 111, 127, 119, 85, and 128 symmetry functions for hydrogen, carbon, nitrogen, oxygen, and sulfur atoms, respectively. Using this set of atomic descriptors in the input layer of committees of eight neural networks, we train two cNN models: one is trained on electronic couplings directly, and the other is trained on the correction to the fitted AOM model (Δ-ML).

In the plots of Figure 6, results for the AOM, the direct learning model (cNN), and the Δ-learning model are shown in violet, blue, and green lines, respectively. The AOM results converge fast when the sampling protocol for AOM fitting is employed. Δ-ML outperforms direct learning in terms of both convergence behavior and accuracy metrics. With less than 100 data points, Δ-ML improves on AOM in both the MAE metric (3 meV lower than AOM) and, most significantly, in the MAX error metric (factor of 2 lower than AOM). However, the Δ-ML estimates have larger errors in the MURE metric compared to AOM. As discussed above, this is due to the fact that small absolute errors translate to large relative errors for couplings that are close to zero. However, since the AOM linear scaling relation passes through the origin by definition, this problem is somewhat alleviated in this method. We find that the large MURE comes from dimers in the D4 cluster (see Figure S8), which have rather small couplings spread around zero. If these dimers are excluded, the MURE drops to less than 50% for both ML methods (Figure 6c, dashed lines in blue and green).

**Figure 7:**
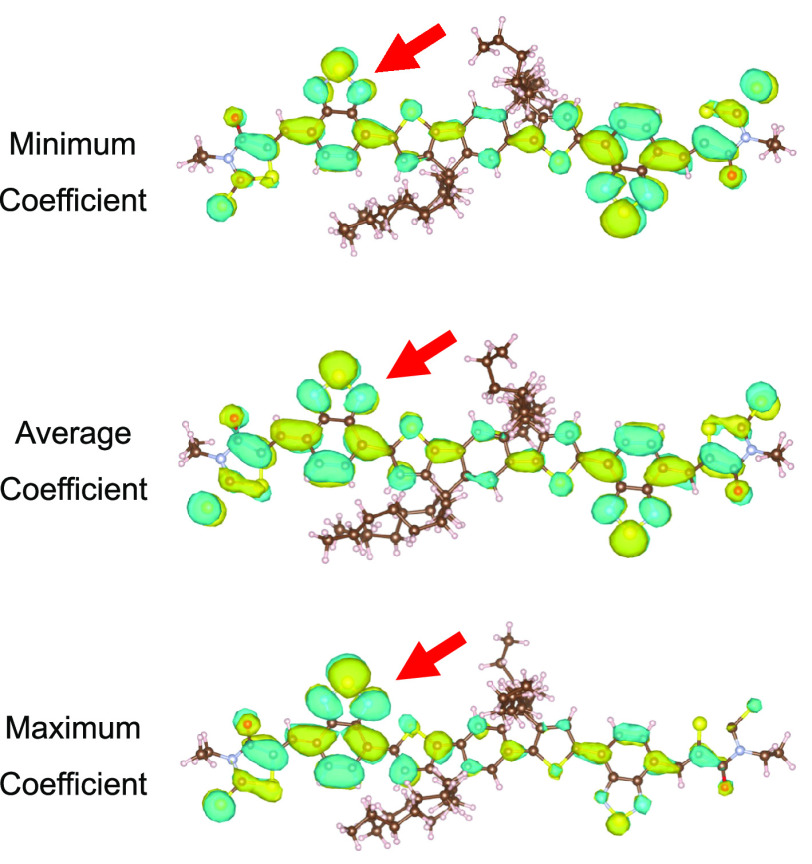
Three cases were the LUMO of an O-IDTBR monomer is localized mostly over the right side (top), both sides (middle), and left side (bottom) of the monomer. Isosurfaces with isovalues of 0.015 are shown and were generated with the VESTA software.^64^

We now would like to explain the large scatter between overlap and electronic coupling shown in Figure 5. The main reason for its limited accuracy is the use of fixed projection coefficients (same set of *c*_*k*_’s in eq 4 for all geometries) in the AOM model. O-IDTBR exhibits strong thermal fluctuations of the dihedral angles that connect the different molecular units, which results in significant variations in the degree of (de)localization of the FMO along the MD trajectory. Representing the FMOs using fixed expansion coefficients may therefore not be as successful for O-IDTBR as for rubrene. We have illustrated three cases in Figure 7 in which the expansion coefficient of an sp^2^ carbon (marked by an arrow) is minimum (0.077), average (0.254), and maximum (0.409). These states correspond to configurations where the FMO (LUMO) of the molecule is preferentially localized on the right-hand side, approximately equally distributed, and preferentially localized on the left-hand side. Obviously, fixed expansion coefficients are no longer a reasonable approximation for this molecule. It results in the very strong scatter shown in Figure 5.

**Figure 8:**
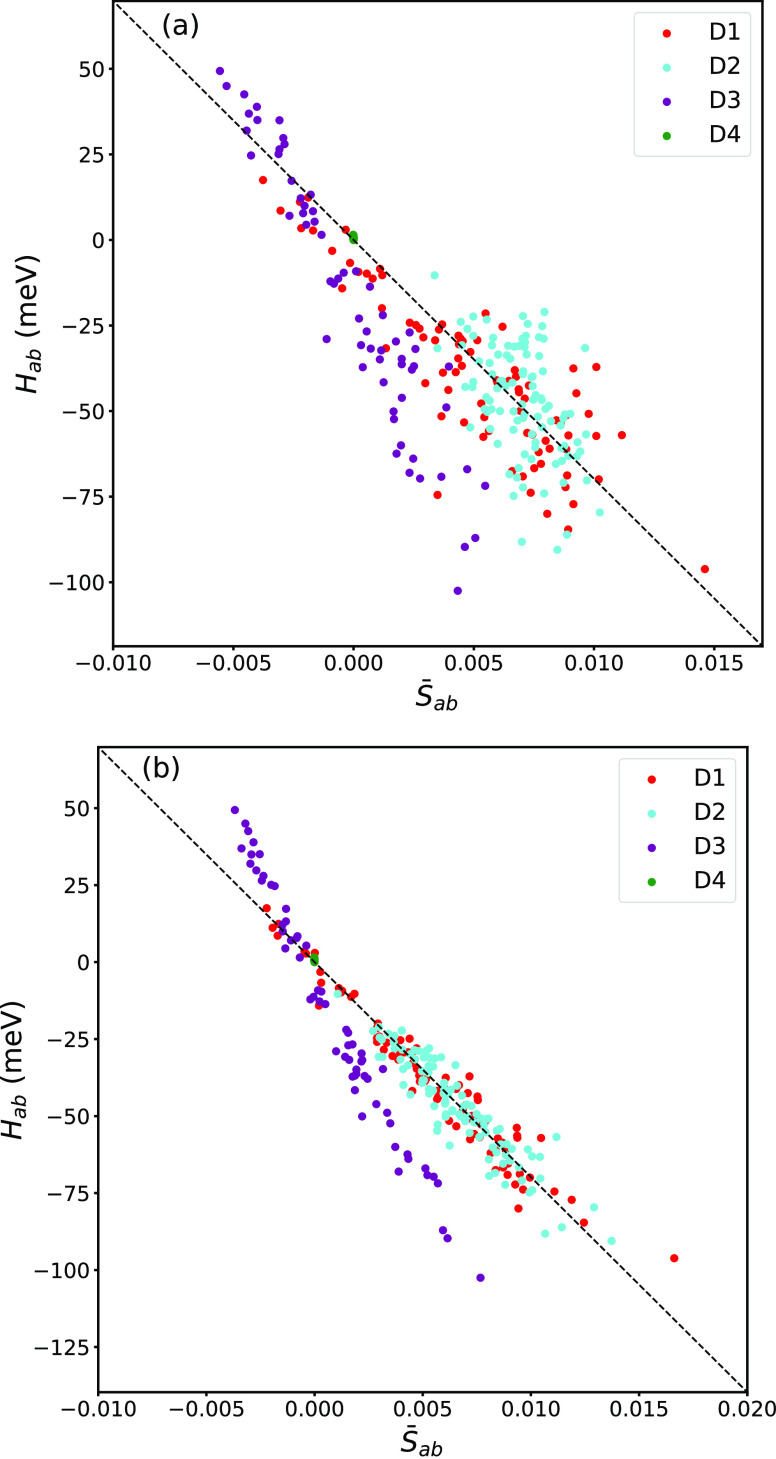
Scatter plots of *H*_*ab*_ vs *S̅*_*ab*_ for O-IDTBR using (a) fixed expansion coefficients for FMO reconstruction (200 data points taken from Figure 5b) and (b) reoptimized expansion coefficients for each data point. Note the reduction in scatter after reoptimization.

In Figure 8, a subset of 200 randomly selected dimers from Figure 5 are replotted (fixed expansion coefficients, panel a) and compared to the results obtained after reoptimization of the expansion coefficients (panel b). Using the optimized expansion coefficients greatly reduces scatter in the data, resulting in errors that are similar to the ones for rubrene. However, in a charge transport simulation, it is not practical to calculate the DFT FMOs of each monomer at each time step due to the high computational cost involved.

Evidently, ML of the effects of variations in the orbital delocalization on electronic coupling is more challenging than the learning of mainly geometry-based changes as in rubrene. This explains the larger errors for O-IDTBR on all error metrics. The proposed ML models are based on the sum of atomic contributions (eq 6), and the environment around each atom is described by neighbors within the cutoff radius. However, while the local environment of the marked atom is very similar in all three cases of Figure 7, the character of the FMO differs substantially. Changes in FMO may be due to long-range effects not well described by short-range symmetry functions. One would require a cutoff radius of at least 30 Å in order to account for such long-range effects. However, the cost of calculating symmetry functions is the bottleneck for the efficiency of ML models, and such large cutoffs would not be practical.

The development of a model to accurately and efficiently estimate expansion coefficients is a promising avenue for further research, based on the observed improvement in results shown in Figure 8.

### Computational Cost

Above we demonstrated that Δ-ML provides a significant improvement to the AOM estimation of electronic couplings. During charge transfer simulations, these estimations are performed millions of times, so their overhead must be kept to a minimum. To benchmark the efficiency of ML models, the calculation time was measured on a single core of an Intel Xeon CPU E5-2650 v4 @ 2.20 GHz. In the case of rubrene, the trained machine learning model requires 40 ms to evaluate electronic coupling for a single dimer, whereas AOM requires 26 ms. This relatively small cost overhead associated with improving accuracy will not be a bottleneck in practical applications. To further improve efficiency, hydrogen atoms can be removed from the current model, since they have very small contribution to the electronic coupling and play a negligible role in describing the local environment in symmetry functions. The removal of hydrogens from rubrene (C_42_H_28_) results in 40% fewer atoms being included in the summations in eq 9 and eq 11 when SFs are calculated, resulting in a 3-fold improvement in efficiency, i.e., 14 ms/prediction, with minimal loss in accuracy (see Figure S3). For O-IDTBR dimers, using a single core, the AOM takes ∼190 ms for a single electronic coupling calculation, whereas the Δ-ML model takes ∼130 ms. Again, the cost overhead of improving the AOM model by ML would not be the bottleneck in practical charge transport simulations.

## Conclusions

We have presented a set of tools that facilitate ultrafast estimation of electronic couplings between molecules, which is necessary for the simulation of charge transport in organic semiconductors. Initially, a sampling protocol was developed by combining a geometrical descriptor, AMD, and farthest point sampling to sample the reference data for fitting AOM models. Using this protocol ensures convergence of AOM fitting with a small number of reference data calculations, eliminating the need for chemical intuition. Various neural network models and sampling methodologies were examined to model the electronic couplings of rubrene dimers, and it was determined that Δ-ML committee neural networks (cNNs) and query by committee (QbC) sampling provided the most accurate results. By exploiting the physics-motivated AOM model as a baseline, the Δ-ML was trained on the difference between AOM and reference data (sPOD/PBE). This approach allows us to achieve similar or better accuracy than previous efforts^37−42^ with training sets that are at least an order of magnitude smaller than those used previously.

With this approach, the accuracy of electronic coupling predictions is improved according to all error metrics employed, at the cost of doubling the computational time. It is important to note, however, that the level of improvement is influenced by the baseline. An AOM model’s cost efficiency comes from its use of fixed FMO projection coefficients for the molecule in question. This is found to be a good approximation in rubrene, where the AOM overlaps exhibit a strongly linear correlation with reference electronic couplings. However, in cases where the FMO character of the molecule changes significantly during dynamics, as in O-IDTBR, the AOM will provide a less accurate baseline. Using an additional machine learning model to estimate the expansion coefficients of the FMOs of molecules will provide higher-quality results and serve as a more reliable baseline for Δ-ML. We are currently working along these lines.

Concerning the active learning method, the use of uncertainty as the metric or score, as in QbC, is very popular; however, other scores can also be utilized. The expected model output change (EMOC) is another score recently adopted in the chemical community.^65^ With this method, AL will select data points with the largest expected change in model output. Considering that the current study does not compare active learning methods and that QbC performs well, such a comparison needs to be conducted in future studies.

Finally, in terms of generalizability of the model, we expect it to be generalizable for cases such as π-conjugated molecules, where the FMO of a molecule can be approximated by its local geometry. When FMOs have nonlocal dependencies, however, a new model must be trained from scratch. Constructing an AOM+Δ-ML correction is straightforward since with the proposed protocol for fitting AOM models and QbC sampling for training ML models, a small number of reference data point calculations and minimum human intervention are required. In addition, newly visited dimer configurations will be detected during the course of the application by examining the standard deviation of ML predictions. The extrapolation occurrence will be handled automatically by retraining the ML model using such data points.

## Data Availability

Sampling and prediction codes can be found in https://github.com/blumberger/ml_electronic_coupling.git.

## Supporting Information Available

Further information about the data sets, clustering, geometry of dimers, and other sampling methods (PDF)
