# Supplementary material for: Ultrafast Electronic Coupling Estimators: Neural Networks versus Physics-Based Approaches
Source: J Chem Theory Comput. 2023 Jun 22;19(13):4232–42. doi: 10.1021/acs.jctc.3c00184 (PMC10339673; doi:10.1021/acs.jctc.3c00184)
Supplement: Supplementary file 1 — ct3c00184_si_001.pdf [file ct3c00184_si_001.pdf]

Supporing Information

Ultrafast electronic coupling estimators: neural networks vs physics-based approaches

Roohollah Hafizi,[a] Jan Elsner,[a] and Jochen Blumberger*[a]

[a] *Department of Physics and Astronomy and Thomas Young Centre, University College London, Gower Street, London WC1E 6BT, United Kingdom*

* **E-mail**: j.blumberger@ucl.ac.uk

1 Rubrene Dataset

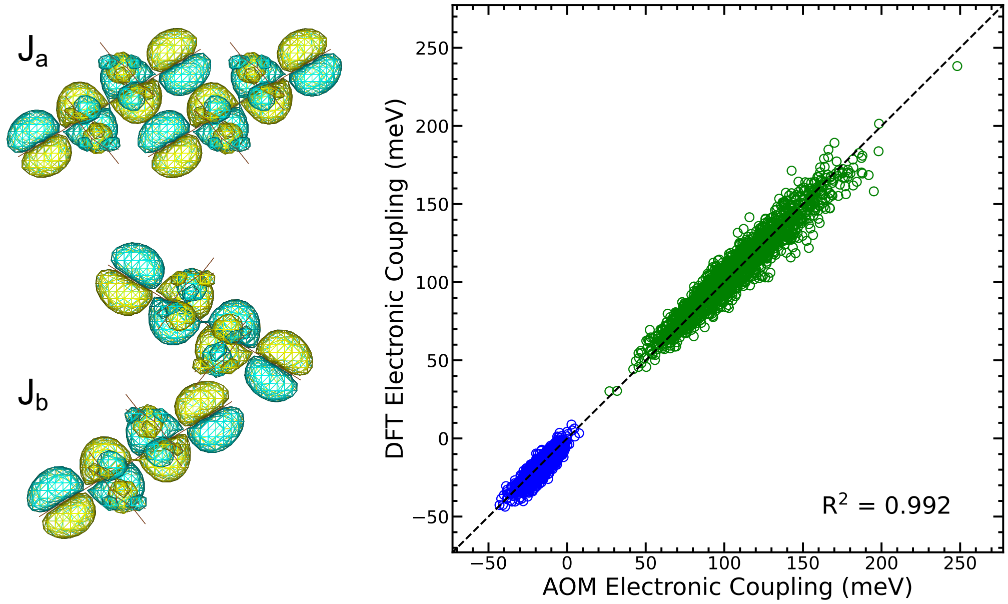

Fig. S1: Orbital isosurfaces of the coupled diabatic states obtained from the sPOD/PBE method for J_a and J_b electronic couplings in rubrene. The right hand side panel shows the correlation between AOM electronic couplings and reference sPOD/PBE values (J_a couplings in green, J_b couplings in blue).

The left-hand side of figure S1 shows the two distinct dimer types in rubrene, J_a and J_b. Isosurfaces of the coupled sPOD/PBE diabatic orbitals are shown in each case, see reference **?** for further details. J_a dimers benefit from greater overlap between orbitals due to close stacking, resulting in larger electronic couplings than for J_b. The right-hand side of figure S1 shows the correlation between AOM electronic couplings and reference sPOD/PBE values. The large value of $R^2 = 0.992$ and small mean absolute error, maximum absolute error and mean relative unsigned error (4.5 meV, 37.0 meV and 14.6 %, respectively) indicate that the AOM is good approximation for this system. In this figure, green circles correspond to J_a couplings, while blue circles correspond to J_b couplings.

2 Parameters of symmetry functions

As a structural descriptor, we employ two types of symmetry functions: radial (G_2) and angular (G_3), respectively. The function forms of each are presented in the main text. Both of these symmetry functions have a cut-off radius of 8 Angstroms. To describe the radial distribution of atoms around a central atom, 8 radial symmetry functions are assigned to each element pair, with the center for each function being selected according to Imbalzano's rule.[?] Accordingly, 8 Gaussian functions are centered at r = [1.0, 1.2968, 1.6818, 2.181, 2.8284, 3.668, 4.7568, 6.1688], with widths (η) of [11.349, 6.7481, 4.0125, 2.3858, 1.4186, 0.8435, 0.5016, 0.2982].

For the angular part, for each element triplet, we describe the radial environment by 2 Gaussains centered at r = [4.0, 5.6569] with widths of η = [0.3643 0.1821]. The parameters are also determined using Imbalzano's rule. Angular distribution of atoms around the central atom is described by two λ's [-1.0, 1.0] and two ζ's [1.0, 6.0]. Accordingly, we have eight angular symmetry functions for each element triplet.

3 Training set sampling: FPS vs. random sampling.

Although being very successful for sampling structures for fitting AOM, Farthest Point Sampling (FPS) is not particularly successful for sampling the training data points for the neural network. In the main text we showed that NN models trained on training sets sampled by random sampling and FPS+AMD sampling vary minimally in terms of the errors of predictions. In figure S2, we show that this is not improved by using symmetry functions instead of AMD i.e. the drawback of FPS for sampling training data for the neural network is not descriptor dependent. Compared to QbC sampling (in the main text), both random and FPS sampling require much more data for convergence of sampling.

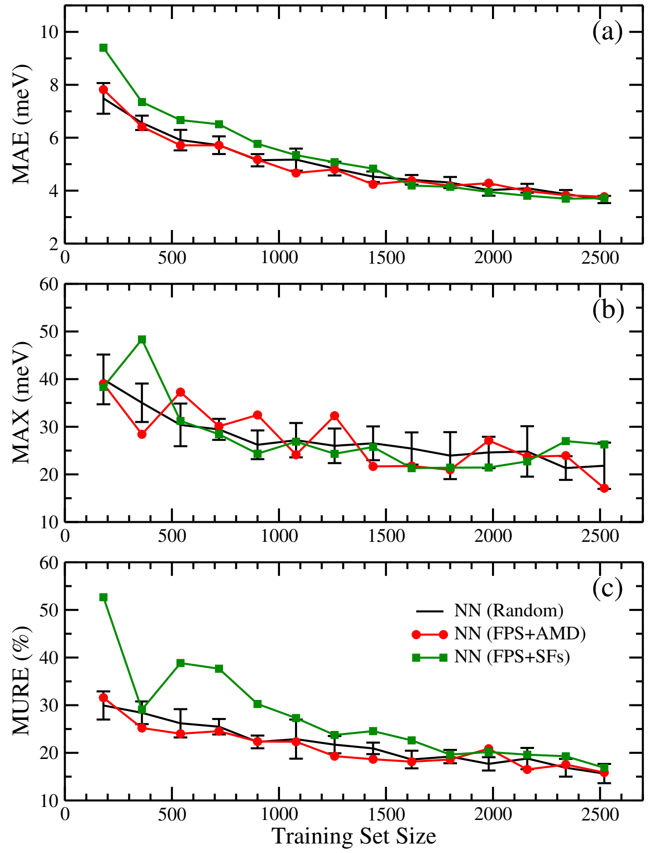

Fig. S2: A comparison of machine learning models of rubrene's electronic coupling based on (a) MAE, (b) MAX, and (c) MURE when the training set is sampled randomly (black), by FPS using AMD structure descriptors (red), and by FPS using symmetry functions (green).

4 Machine learning models without hydrogen atoms

The calculation of symmetry functions is the bottleneck of efficiency when making predictions with NNPs. Due to the fact that hydrogen atoms contribute minimally to the electronic coupling of molecular dimers, it is possible to reduce the cost of SF calculations by removing hydrogen atoms:

1. There will be a reduction in the number of atoms that are checked to see if they are inside the cutoff radius.

2. Since symmetry functions are calculated separately for element pairs (radial) and element triplets (angular), removing atoms reduces the amount of calculations required.

For instance, in the case of rubrene, removing hydrogen atoms reduces the total number of atoms in the dimer from 140 to 84. The distance matrix is therefore reduced by a factor of $(84/140)^2 = 0.36$. In addition, only CC (radial) and CCC (angluar) symmetry functions have to be calculated now, resulting in three times less SFs to be evaluated. Figure S3 illustrates the achievable accuracy when hydrogens are removed from rubrene dimers' descriptions. It is evident that with considerable cost savings, accuracy is minimally affected.

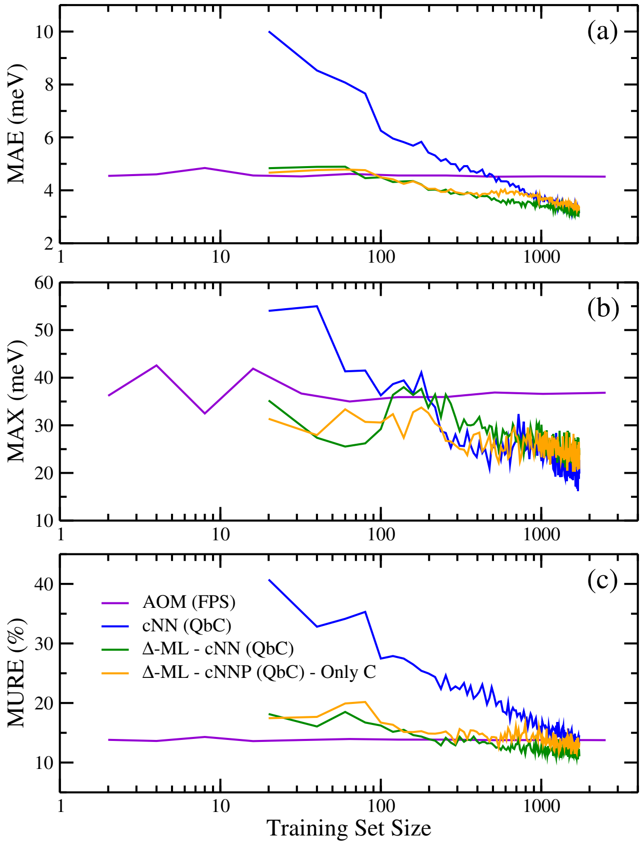

Fig. S3: A comparison of Δ-ML models of rubrene's electronic coupling based on (a) MAE, (b) MAX, and (c) MURE when hydrogen atoms are considered in (green) / removed from (orange) description of the local environment. The data relating to the AOM model (violet) and the direct model (blue) are also included for comparison.

5 O-IDTBR Dimers

5.1 Orientation of dimers in the dataset

5770 O-IDTBR dimer pairs are taken from classical molecular dynamics trajectories using a force field specifically parameterized for the family of IDTBR nonfullerene acceptors[?]. These dimers have 4 distinct orientations as shown in figure S4.

5.2 O-IDTBR clusters

We evaluated the quality of the AMD descriptor in capturing the underlying order of O-IDTBR dimers. HDBSCAN and the Euclidian distance metric between AMD descriptor vectors were used for this purpose. In figure S5, we can see that there are four types of dimers (D1, D2, D3, and D4) in the data set. They are displayed in red, blue, purple, and green, respectively. As shown in figure S5, clustering

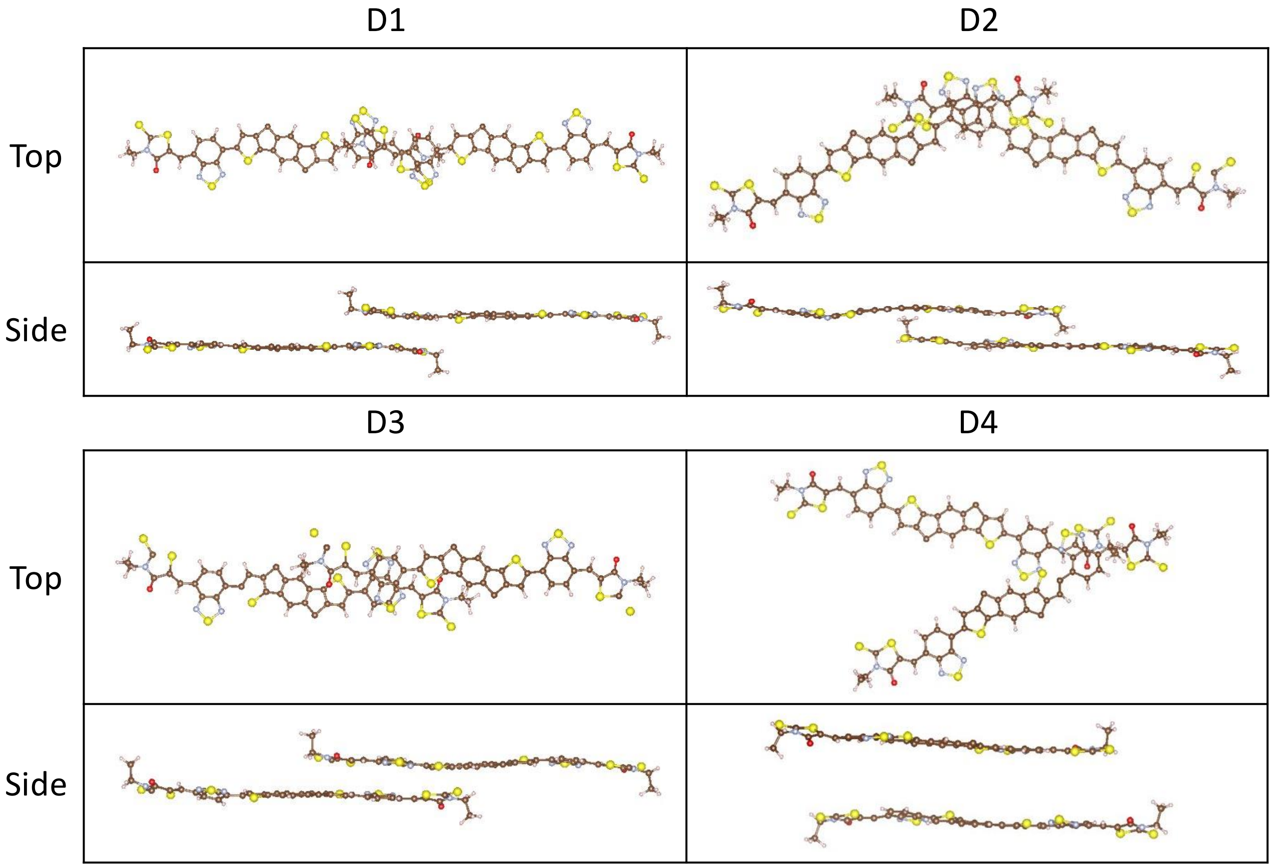

Fig. S4: Four O-IDTBR dimers with significant electronic coupling values between monomers. These are the only dimer types used to train and test the model.

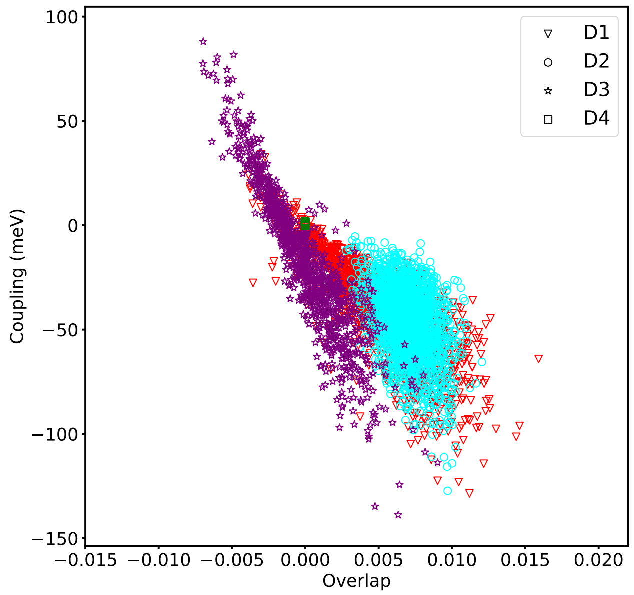

Fig. S5: Clustering of the O-IDTBR dataset using HDBSCAN and AMD descriptor: The reference data contains four clusters as indicated by the four different colors in Figure 5 of the main text. After clustering, four clusters of data are correctly detected, indicated by circles, triangles, squares, and stars. Clusters are detected without error or unclustered data points.

resulted in four clusters indicated by triangles, circles, stars, and squares. All dimers are clustered correctly and no dimers remain unclustered.

5.3 AOM model of O-IDTBR

The AOM model was fitted using the fitting protocol described in the main text. A converged $\bar{C}$ value of -7070 meV was obtained after adding 200 dimers to the training set (see figure S7a). Figure S6 clearly shows that the correlation between the AOM and reference couplings (R^2=0.67) is less strong than for rubrene (R^2=0.99). The model results in a MAE of 12.4 mev (fig. S7b, green), a maximum absolute error of 94 meV (fig. S7c), and a mean unsigned relative error of 62 % (fig. S7d, red) on the test set. It must be noted that the reference values for D4 dimers are close to zero. Consequently, when making predictions on D4 dimers, the MRUE will be large, since small absolute errors translate into large relative errors. Because the assumed AOM relation Eqn 5 of the main text passes through the origin, the MAE of prediction using the AOM model benefits from including D4 dimers in dataset; however, MRUE gets worse. Since the magnitude of deviation from a linear relationship is more pronounced far from the origin, D4 dimers do not affect MAX error. As shown in figure S7, when the D4 dimers are removed from the test set, the MAE increases to 14.3 meV, and the MRUE decreases to 56 %.

5.4 O-IDTBR prediction on clusters

For the purpose of illustrating the effect of small electronic couplings values on the statistics, especially the MRUE, we have plotted the predictions of Δ-ML model on the four clusters of O-IDTBR in the test set in Figure S8. Although the MAE and MAX of predictions on cluster D4 are smaller, the relative error of predictions on this cluster is significantly higher than those on the other clusters. The dashed

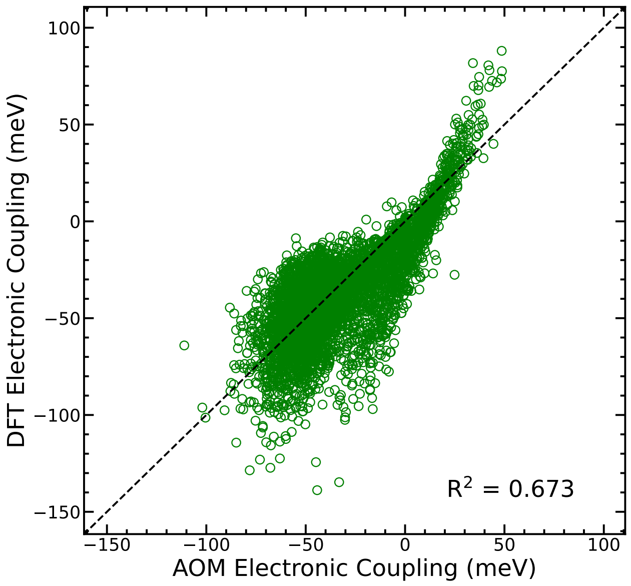

Fig. S6: Correlation between AOM electronic couplings and reference sPOD/DFT values for the O-IDTBR dataset.

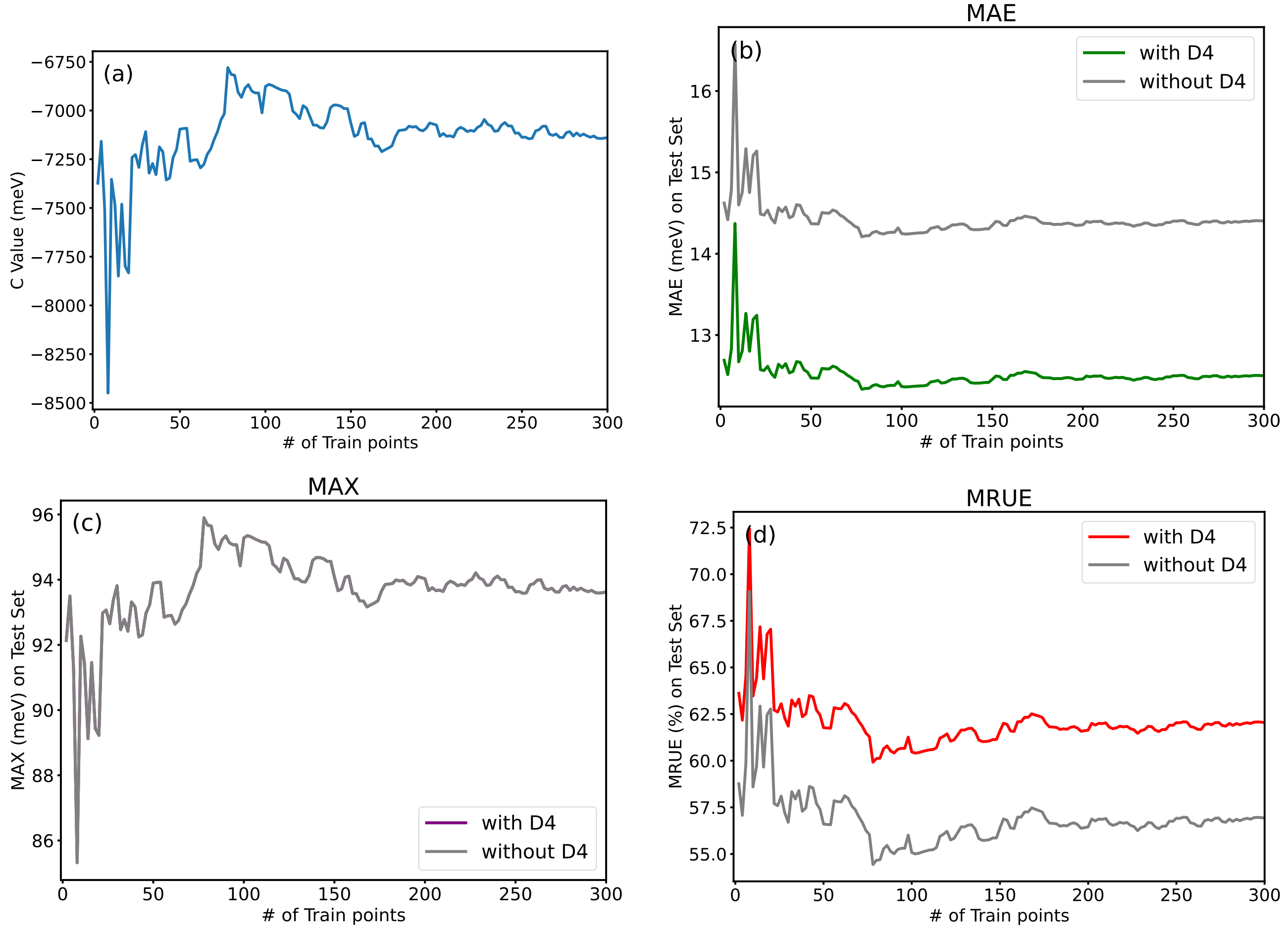

Fig. S7: AOM on O-IDTBR

lines in Figure 6 of the main text represent the errors obtained after the removal of D4 dimers. The MAE of the AOM predictions, as well as that of the ML models, increases; however, the increase in the value of the AOM is markedly greater than the increase in the value of the ML models. Due to the removal of data points with small values, the MAX error remains unchanged. When the D4 cluster is removed from the test, MRUE drops on all models; for the AOM model, it decreases from 62% to 57%. Since other models have a much larger MAE value, this small change is not noticeable in Figure 6 of the main text, but it is clearly visible in Figure S7 of the SI. The MRUE of both ML models drops dramatically from $\simeq$250% on average to less than 50%. In particular, the Δ-ML-cNN can achieve a MRUE of 40%, which is 17% lower than the MRUE achieved by the AOM. Accordingly, excluding the D4 clusters from the test set would have a greater benefit for ML models than AOM, so with just 200 data points the Δ-ML-cNN model would outperform AOM.

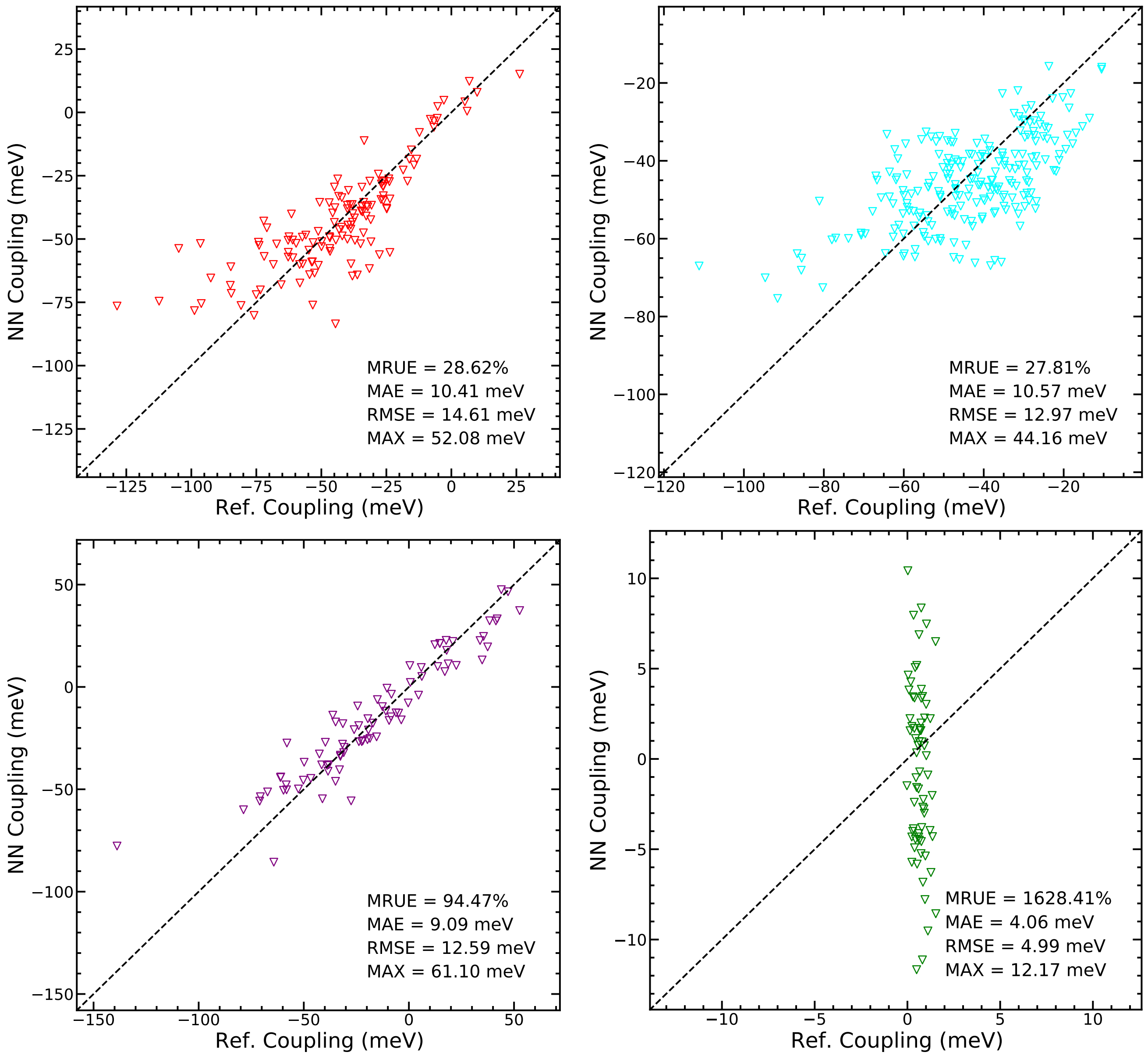

Fig. S8: Prediction of the Δ-ML model on clusters of O-IDTBR dataset. (red) D1, (cyan) D2, (purple) D3, and (green) D4.
